# Poor outcome in hypoxic endometrial carcinoma is related to vascular density

**DOI:** 10.1038/s41416-019-0461-2

**Published:** 2019-04-23

**Authors:** Casper Reijnen, Willem Jan van Weelden, Martijn S. J. P. Arts, Johan P. Peters, Paul F. Rijken, Koen van de Vijver, Maria Santacana, Peter Bronsert, Johan Bulten, Marc Hirschfeld, Eva Colas, Antonio Gil-Moreno, Armando Reques, Gemma Mancebo, Camilla Krakstad, Jone Trovik, Ingfrid S. Haldorsen, Jutta Huvila, Martin Koskas, Vit Weinberger, Lubos Minar, Eva Jandakova, Marc P. L. M. Snijders, Saskia van den Berg-van Erp, Heidi V. N. Küsters-Vandevelde, Xavier Matias-Guiu, Frederic Amant, ENITEC-consortium, Leon F. A. G. Massuger, Johan Bussink, Johanna M. A. Pijnenborg

**Affiliations:** 1 Department of Obstetrics and Gynaecology, Radboud Institute for Health Sciences, Radboud University Medical Center, Nijmegen, The Netherlands; 2 Department of Obstetrics and Gynaecology, Canisius-Wilhelmina Hospital, Nijmegen, The Netherlands; 3 Department of Radiation Oncology, Radboud University Medical Center, Nijmegen, The Netherlands; 4 Department of Pathology, Ghent University Hospital, Cancer Research Institute Ghent (CRIG), Ghent, Belgium; 5 Department of Pathology and Molecular Genetics and Research Laboratory, Hospital Universitari Arnau de Vilanova, University of Lleida, IRBLleida, CIBERONC, Lleida, Spain; 6 Institute of Pathology, University Medical Center, Freiburg, Germany; 7 Department of Pathology, Radboud University Medical Center, Nijmegen, The Netherlands; 8 Department of Obstetrics and Gynecology, University Medical Center, Freiburg, Germany; 9 German Cancer Consortium, German Cancer Research Center, Heidelberg, Germany; 10 Biomedical Research Group in Gynecology, Vall Hebron Institute of Research, Universitat Autònoma de Barcelona, CIBERONC, Barcelona, Spain; 11 Gynecological Department, Vall Hebron University Hospital, CIBERONC, Barcelona, Spain; 12 Pathology Department, Vall Hebron University Hospital, CIBERONC, Barcelona, Spain; 13 Department of Obstetrics and Gynecology, Hospital del Mar, PSMARB, Barcelona, Spain; 14 Department of Obstetrics and Gynecology, Haukeland University Hospital, Bergen, Norway; 15 Centre for Cancer Biomarkers, Department of Clinical Science, University of Bergen, Bergen, Norway; 16 Mohn Medical Imaging and Visualization Centre, Department of Radiology, Haukeland University Hospital, Bergen, Norway; 17 Department of Pathology, University of Turku, Turku, Finland; 18 Obstetrics and Gynecology Department, Bichat-Claude Bernard Hospital, Paris, France; 19 Department of Gynecology and Obstetrics, Faculty of Medicine, Masaryk University, Brno, Czech Republic; 20 Institute of Pathology, Faculty of Medicine, Masaryk University, Brno, Czech Republic; 21 Department of Pathology, Canisius-Wilhelmina Hospital, Nijmegen, The Netherlands; 22 Department of Oncology, KU Leuven, Leuven, Belgium; 23 Department of Gynaecologic Oncology, Center for Gynaecologic Oncology, Amsterdam, The Netherlands

**Keywords:** Prognostic markers, Endometrial cancer

## Abstract

**Background:**

Identification of endometrial carcinoma (EC) patients at high risk of recurrence is lacking. In this study, the prognostic role of hypoxia and angiogenesis was investigated in EC patients.

**Methods:**

Tumour slides from EC patients were stained by immunofluorescence for carbonic anhydrase IX (CAIX) as hypoxic marker and CD34 for assessment of microvessel density (MVD). CAIX expression was determined in epithelial tumour cells, with a cut-off of 1%. MVD was assessed according to the Weidner method. Correlations with disease-specific survival (DSS), disease-free survival (DFS) and distant disease-free survival (DDFS) were calculated using Kaplan–Meier curves and Cox regression analysis.

**Results:**

Sixty-three (16.4%) of 385 ECs showed positive CAIX expression with high vascular density. These ECs had a reduced DSS compared to tumours with either hypoxia or high vascular density (log-rank *p* = 0.002). Multivariable analysis showed that hypoxic tumours with high vascular density had a reduced DSS (hazard ratio [HR] 3.71, *p* = 0.002), DDFS (HR 2.68, *p* = 0.009) and a trend for reduced DFS (HR 1.87, *p* = 0.054).

**Conclusions:**

This study has shown that adverse outcome in hypoxic ECs is seen in the presence of high vascular density, suggesting an important role of angiogenesis in the metastatic process of hypoxic EC. Differential adjuvant treatment might be indicated for these patients.

## Background

Most endometrial carcinoma (EC) patients present with early-stage disease and have a favourable outcome. Nevertheless, 15% of all patients suffer from recurrent disease and subsequently have a poor outcome.^1–3^ Approximately half of these recurrences occur in patients primarily diagnosed with low-risk EC.^1,4^ Improved identification of patients at high risk for recurrence is crucial to prevent both over- and undertreatment.

Hypoxia is known to be an important feature of aggressive EC and drives metastatic potential.^5–8^ When solid tumours outgrow their vasculature beyond the size of 0.1 mm^3^, hypoxia may occur.^9^ As a response to chronic hypoxia, tumour cells will activate genes associated with more aggressive phenotype and resistance to chemotherapy and radiotherapy.^10^ Hypoxia-inducible factor 1 (HIF-1), formed after heterodimerisation of its subunits HIF-1α and HIF-1β, plays a key role in this process.^11,12^ HIF-1 activates downstream genes that enhance cell survival by maintaining intracellular pH, stimulating angiogenesis to increase oxygen delivery and switching to anaerobic glycolysis.^12,13^ More specifically, an important downstream target is carbonic anhydrase 9 (CA9), whose encoded protein, carbonic anhydrase IX (CAIX), regulates intracellular pH by converting carbon dioxide to carbonic acid.^14^ By adaptation of tumour cells to a hostile microenvironment, tumour proliferation can commence even in hypoxic areas.^15^ Also in normoxic conditions, HIF-1 can be activated; however, downstream activation is present in lesser extent.^16,17^ In this perspective CAIX expression, one of the key effector proteins of HIF-1, has been shown to be more specifically related to hypoxia and poor outcome.^18^

Next to maintenance of intracellular pH, stimulation of angiogenesis is an important response to hypoxia. Vascular endothelial growth factor (VEGF), another downstream target of HIF-1, is also correlated with hypoxia and angiogenesis in several cancer types, including EC.^19–22^ Angiogenesis can be assessed by microvessel density (MVD) and is prognostically associated with deep myometrial invasion (MI), lymphovascular space invasion (LVSI) and poor overall survival in EC.^23^ Although earlier studies suggest correlation between hypoxia, angiogenesis and poor outcome, the prognostic value has not yet been studied before.^5,6^ Therefore, we have investigated the prognostic value of hypoxia and angiogenesis in EC, assessed with CAIX expression and MVD.

## Methods

### Patients

Data and tumour slides were collected previously for a study analysing the value of L1CAM expression in ECs, which included ECs from 11 collaborating European Network for Individualised Treatment of Endometrial Cancer (ENITEC) centres.^24,25^ Only cases diagnosed by an expert gynaecological pathologist, with complete data on treatment and pathological examination and at least 36 months of follow-up, were included. Cases with a non-endometrioid component were categorised as non-endometrioid. The 1199 cases included in the original study were randomly selected using SPSS version 22 (SPSS IBM, New York, NY, USA), resulting in a database of 403 patients for the present study. These cases were not statistically different from the original cases for the most important baseline characteristics.

### Tissue and staining

Four micrometre sections, derived from formalin-fixed, paraffin-embedded ECs were used to visualise CAIX and blood vessels. Sections were mounted on Superfrost slides (Menzel–Gläser). Slides were deparaffinated in Histochoise (VWR H103-4L) and rehydrated (graded ethanol: 100–96–70% and de-ionised water). Next, citrate buffer antigen retrieval was performed for 30 min (Target retrieval solution 10×, pH 6 citrate, Dako Cytomation, 96 °C). Prior to incubation with the primary antibodies, sections were blocked with 5% normal goat serum (Jackson ImmunoResearch) in primary antibody diluent (PAD, BIORAD BUF014), 30 min at room temperature. Thereafter, sections were co-stained for CAIX (Novus Biologicals NB100-417, 1:100) and vessels (CD34, ABCAM ab8536, 1:300), 60 min at 37 °C. Secondary incubation was performed using Cy^TM^3 Fab Fragment Goat Anti-Rabbit immunoglobulin G (IgG) (H + L) polyclonal IgG (Jackson ImmunoResearch 111-167-003) for CAIX and CF^®^488a Goat anti-Mouse IgG (H + L), F(ab′)2 fragment polyclonal IgG (Biotium CF488A) for the vessels, 60 min at 37 °C. All antibodies were diluted in PAD. In between stainings, sections were rinsed with phosphate-buffered saline (JT Baker 4391.9010). 4′,6-Diamidino-2-phenylindole (DAPI) (Santa Cruz Biotechnology AB-17.0097) was used as a counterstain to stain all nuclei, and finally the sections were mounted with Fluoromount W (Serva 21634.01). Haematoxylin and eosin staining was used for morphological evaluation. CAIX expression was scored as the fraction of epithelial tumour cells with positive membranous staining.

### Image analysis

Tumour slides were analysed using a digital image analysis system after scanning of the whole slides with the Axio Imager D2 microscope (Carl Zeiss, GmbH, Oberkochen, Germany) using a Prior lumen 200 metal halide lamp (Prior Scientific, Rockland, USA), Axiocam 503 mono 16-bit camera (1936 × 1460 pixel, Carl Zeiss, GmbH) and a computer-controlled motorised stage (Carl Zeiss, GmbH) directed by Zen Pro software (Carl Zeiss, GmbH).^26^ Each slide was scanned for three signals: DAPI (all nuclei), Alexa488 (CD34) and Cy3 (CAIX), by means of a 10× objective using standardised shutter times for each signal (1, 25 and 50 ms, respectively). After scanning, grey-scale images of all three recorded signals were used for analysis.

For analysis of CAIX staining, only membranous expression on epithelial tumour cells was analysed. Areas of necrosis, large vessels and tumour stroma, determined using H&E-stained adjacent tumour slides, were therefore manually excluded from the analysis (i-Vision for Mac; BioVision Technologies, Exton, PA, USA). Next, thresholds for segmentation of the fluorescent signals were interactively set above the background staining for each individual marker and adjusted for each sample in order to optimise the signal to background ratio using ImageJ software (Wayne Rasband, National Institute of Mental Health, National Institutes of Health). An interactively set threshold limits inter-sample variability by correction for differences in immunofluorescence staining intensity.^26,27^ The resulting binary images were used to calculate the fraction of CAIX (fCAIX) relative to the total tumour area. To minimise bias of non-specific staining, only positive signals exceeding 5 pixels were included.

The MVD was measured according to the Weidner method.^28^ In short, surrounding epithelial tumour cells three areas with the highest density of vessels were selected by the assessor (M.A.) using a ×200 magnification. To correct for objects that exceed the image borders, only objects exceeding the left and upper border were included. To minimise bias of non-specific staining, only positive signals exceeding 2 pixels were included.

**Fig. 1:**
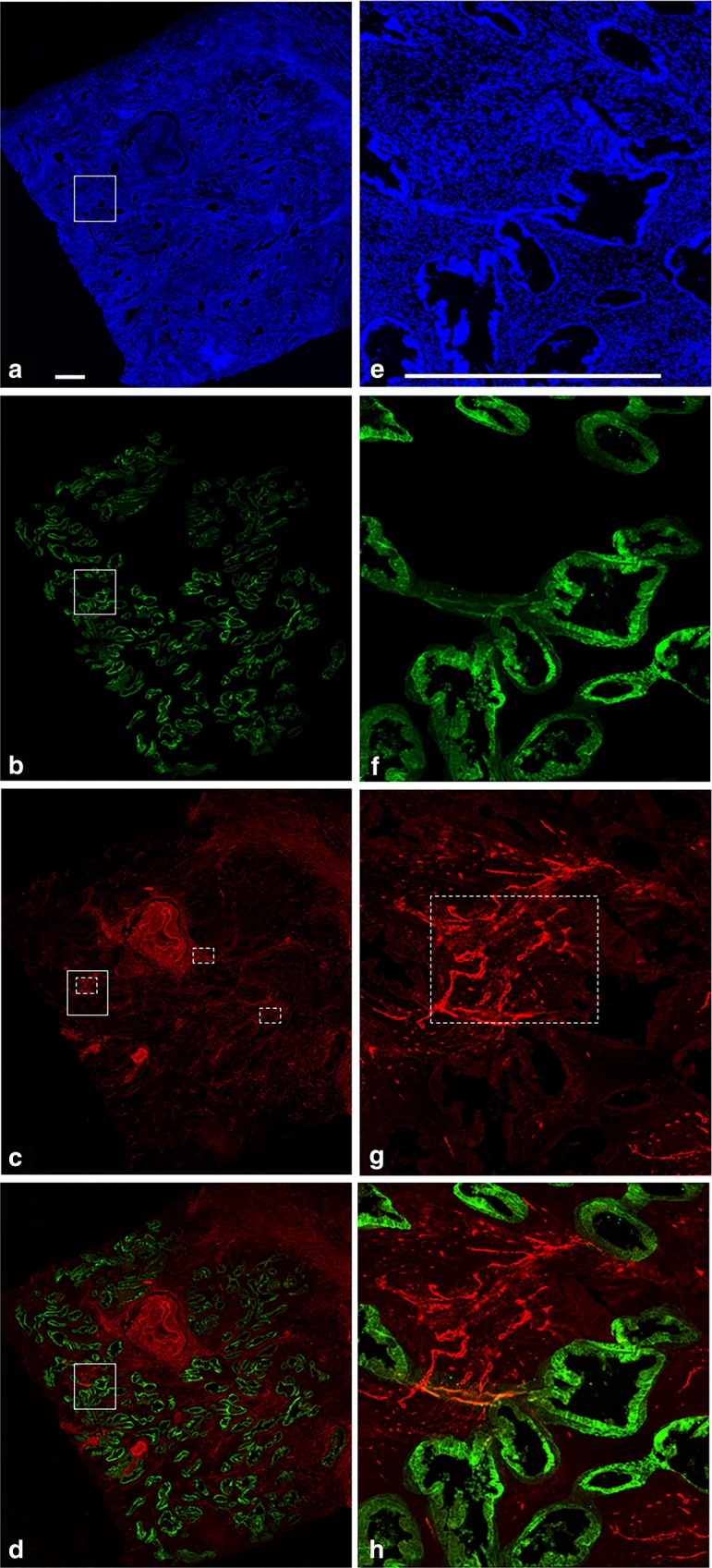
Example of carbonic anhydrase IX (CAIX) and CD34 staining in endometrial cancer. **a** Nuclear 4′,6-diamidino-2-phenylindole (DAPI) staining (blue) for visualisation of tumour nuclei. **b** CAIX staining (green) adjusted for total tumour area, meaning that only epithelial tumour cells were included in the analysis: other tissue, including stroma, necrosis and vasculature has manually been removed and is coloured black by the analysis software (see “Methods” section). **c** CD34 staining of vasculature (red) with three hotspots according to the Weidner method, marked with the interrupted lines. **d** Combined CAIX staining and CD34 staining. **e**–**h** represent representative high-magnification images of the boxed areas in **a**, **b**, **c** and **d**, respectively. Scale bar = 0.5 mm

CAIX expression was considered positive when the fCAIX was above 1%.^29,30^ The MVD was dichotomised over the median. A representative example of CAIX and MVD staining is shown in Fig. 1.

### Statistical analyses

Clinicopathological differences between subgroups were compared with the *χ*^2^ and Fisher’s exact tests for categorical data and the Mann–Whitney *U* test for continuous variables.

Kaplan–Meier curves were constructed for disease-specific survival (DSS), disease-free survival (DFS) and distant-DFS (DDFS). The association between CAIX and MVD and DSS, DFS and DDFS was determined using Cox regression analysis. DSS was calculated from the date of primary treatment to the date of death caused by the disease or, for surviving patients, to the date of the last follow-up. DFS and DDFS were defined as the length of follow-up, after completion of the primary treatment, during which women survived without any clinical sign of (distant) disease recurrence. Distant recurrence included metastases in distant organs and para-aortic lymph nodes. Features identified by univariable regression analysis with *p* < 0.20 were used for multivariable regression analysis. LVSI was coded as negative in case of missing data (*n* = 108) since only substantial LVSI was recently reported as relevant for prognosis of EC. If LVSI was not reported in the pathological report, it was therefore assumed that LVSI was absent.^31,32^ *P* values <0.05 were considered to indicate a significant difference. SPSS version 25 (SPSS IBM, New York, NY, USA) statistical software was used to perform the statistical analyses.

## Results

### Patients

After staining for CAIX and CD34, 18 of the 403 patients were excluded due to insufficient tumour tissue (*n* = 9) and excess of non-specific background staining (*n* = 9). Clinicopathological characteristics of the 385 patients included for analysis are shown in Table 1. Overall, the median age was 64 years and the median follow-up time was 58 months. Of all patients alive at the end of follow-up, 99% had a follow-up of at least 36 months. A total of 67 patients (17.4%) were diagnosed with high-grade EC, including 13 non-endometrioid endometrial carcinomas (NEECs) (3.4%). In total, 106 (27%) EC patients had positive CAIX expression. Forty-seven patients (12.2%) recurred and 21 patients (5.5%) died due to the disease. Of all the patients with recurrence, 14 (3.6%) had a local recurrence, 16 (4.2%) a regional recurrence and 31 (8.1%) a distant recurrence.

**Table 1:** Baseline characteristics of all included patients, associated with CAIX combined with vascular density

Variable	All (*n* = 385)	Not CAIX >1% and high vascular density (*n* = 322)	CAIX >1% and high vascular density (*n* = 63)	P*
Age (years)^a^	64.0 (34.0–89.0)	64.3 (34.0–89.0)	63.5 (43.0–88.0)	0.634
Follow-up (months)^a^	65.1 (0.0–156.0)	63.8 (0.0–156.0)	71.6 (0–148.0)	0.125
Grade				
Low	318 (82.6)	270 (83.9)	48 (76.2)	0.142
High	67 (17.4)	52 (16.1)	15 (23.8)	
Histology				
EEC	372 (96.6)	314 (97.5)	58 (92.1)	0.028
NEEC	13 (3.4)	8 (2.5)	5 (7.9)	
FIGO stage				
I–II	363 (93.8)	305 (94.7)	58 (92.1)	0.406
III–IV	22 (5.7)	17 (5.3)	5 (7.9)	
Myometrial invasion				
<50%	258 (67.2)	218 (67.9)	40 (63.5)	0.494
≥50%	126 (32.8)	103 (32.1)	23 (36.5)	
LVSI^b^				
No	243 (62.8)	200 (90.1)	43 (84.3)	0.234
Yes	30 (7.8)	22 (9.9)	8 (15.7)	
Lymph nodes^c^				
No metastasis	263 (68.0)	223 (95.7)	40 (95.2)	0.891
Metastasis	12 (3.1)	10 (4.3)	2 (4.8)	
Adjuvant treatment				
No	154 (40.0)	143 (44.8)	22 (34.9)	
Radiotherapy	200 (51.9)	161 (50.0)	39 (61.9)	0.084
Chemotherapy	31 (8.1)	18 (5.6)	2 (3.2)	0.429
Recurrence				
No	338 (87.8)	289 (89.8)	49 (77.8)	
Yes	47 (12.2)	33 (10.2)	14 (22.2)	0.008
Local	14 (3.6)	12 (3.7)	2 (3.2)	0.830
Regional	16 (4.2)	13 (4.0)	3 (4.8)	0.792
Distant	31 (8.1)	19 (5.9)	12 (19.0)	<0.001
Death				
No	335 (87.0)	284 (88.2)	49 (77.8)	
Yes	50 (13.0)	38 (11.8)	14 (22.2)	0.027
EC-related	21 (5.5)	12 (3.7)	11 (17.5)	<0.001

*CAIX* carbonic anhydrase IX, *EEC* endometrioid endometrial carcinoma, *NEEC* non-endometrioid endometrial carcinoma, *FIGO* International Federation of Gynaecology and Obstetrics, *LVSI* lymphovascular space invasion, *EC* endometrial carcinoma

*P value of the Mann–Whitney *U* test for continuous, and *χ*^2^ test and Fisher’s exact for categorical variables

^a^Median values (range)

^b^Based on 273 patients

^c^Based on 275 patients

### CAIX expression and MVD

A total of 63 carcinomas (16.4%) showed a positive membranous epithelial CAIX expression and high degrees of vascular density, defined as a MVD above the median (Table 1). CAIX expression with high vascular density was correlated with non-endometrioid histology (7.9% vs. 2.5%, *p* = 0.028), but not with other clinicopathological features. Patients with CAIX-positive ECs and high vascular density experienced more recurrences (22.2% vs. 10.2%, *p* = 0.008) and specifically more distant recurrences (19.0% vs. 5.9%, *p* < 0.001), as well as higher overall mortality (22.2% vs. 11.8%, *p* = 0.027) and EC-related mortality (17.5% vs. 3.7%, *p* < 0.001) (Table 1).

**Fig. 2:**
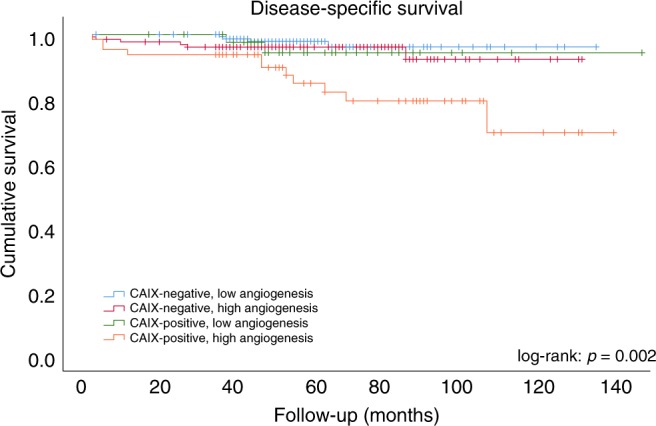
Disease-specific survival (DSS) by carbonic anhydrase IX (CAIX) expression combined with degree of angiogenesis. Log-rank test was used to compare groups

**Fig. 3:**
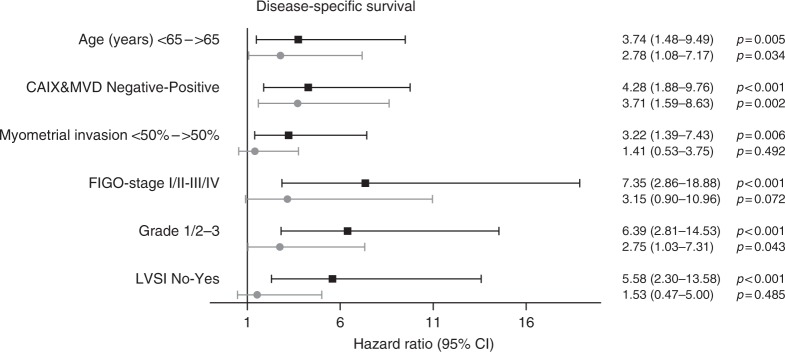
Univariable and multivariable Cox regression analysis of clinicopathological parameters including carbonic anhydrase IX (CAIX) combined with vascular density for disease-specific survival (DSS). The hazard ratios with 95% confidence intervals are depicted by the black line. All risk factors significantly associated with DSS in univariable analysis were included in the multivariable Cox regression analysis, depicted by the grey lines

Figure 2 shows that CAIX expression with high vascular density was associated with a worse DSS compared to CAIX expression with low vascular density and negative CAIX expression (*p* = 0.002). Interestingly, CAIX-positive ECs with low vascular density had a similar outcome as CAIX-negative ECs. Univariable Cox regression analysis revealed that age, CAIX expression with high vascular density, myocardial infarction (MI), International Federation of Gynaecology and Obstetrics (FIGO) stage, grade and lymphovascular space invasion (LVSI) were significantly associated with DSS (Fig. 3). In multivariable analysis, high age, CAIX expression with high vascular density and tumour grade 3 remained significantly associated with reduced DSS, with CAIX and MVD as the most significant parameter (hazard ratio [HR] 3.71, 95% confidence interval (CI) 1.59–8.63, *p* = 0.002).

**Fig. 4:**
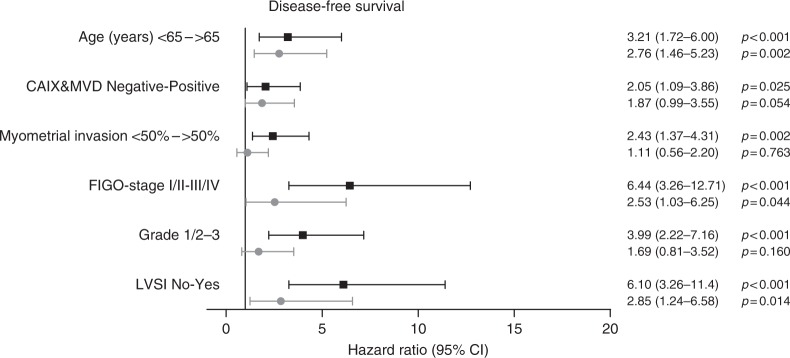
Univariable and multivariable Cox regression analysis of clinicopathological parameters including carbonic anhydrase IX (CAIX) combined with vascular density for disease-free survival (DFS). All risk factors significantly associated with DFS in univariable analysis were included in the multivariable Cox regression analysis, depicted by the grey lines

**Fig. 5:**
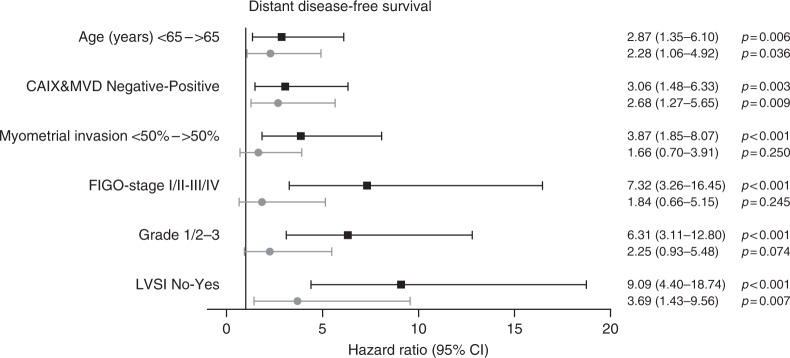
Univariable and multivariable Cox regression analysis of clinicopathological parameters including carbonic anhydrase IX (CAIX) combined with vascular density for distant disease-free survival (DDFS). All risk factors significantly associated with DDFS in univariable analysis were included in the multivariable Cox regression analysis, depicted by the grey lines

Multivariable analysis showed that age, FIGO stage and LVSI were significantly associated with DFS. CAIX expression with high vascular density was nearly significant (HR 1.87, 95% CI 0.99–3.55, *p* = 0.054, Fig. 4). Multivariable analysis for DDFS showed that LVSI and CAIX expression with high vascular density was significantly associated with a reduced DDFS (CAIX and MVD: HR 2.68, 95% CI 1.27–5.65, *p* = 0.009, Fig. 5).

### Individual contribution of CAIX and MVD

Positive CAIX expression was associated with high tumour grade, non-endometrioid histology, higher median MVD and treatment with radiotherapy. In multivariable analysis, CAIX and grade were significantly associated with DSS (HR 2.45, 95% CI 1.05–5.73, *p* = 0.039) (Supplementary Tables 1 and 2). High MVD was correlated with deep MI, but not with other clinicopathological factors. In multivariable analysis, high MVD remained an independent predictor of reduced DSS (HR 2.92, 95% CI 1.13–7.54, *p* = 0.027) (Supplementary Tables 3 and 4). Continuous scoring of CAIX expression showed a significant correlation with DSS as well (data not shown).

## Discussion

In the present study, we have investigated the prognostic value of angiogenesis and hypoxia, assessed with MVD and CAIX expression. We hypothesised that angiogenesis would facilitate haematogenous spread of hypoxic tumour cells with subsequent poor clinical outcome. Additionally, we assumed that this would specifically be facilitated in hypoxic ECs, because of activation of intracellular pathways that induce an aggressive and metastatic phenotype. We have shown that CAIX expression with high vascular density is associated with reduced DSS and DDFS. Interestingly, CAIX-positive ECs with low vascular density had a similar outcome as CAIX-negative ECs. Finally, multivariable analyses for CAIX expression and vascular density showed that both were independent prognostic markers as well.

This is the largest study to date studying CAIX in EC. In contrast to previous studies in EC, we did find significant correlations between CAIX expression and poor outcome, especially in case of high vascular density. Seeber et al.^29^ included 93 patients and found CAIX expression in 76% of ECs.^29^ In this study, no correlation between CAIX expression and outcome was found; however, small sample size and different cut-off value (all degrees of positive staining were regarded as positive) could explain why no correlation was found. Similarly, Pijnenborg et al.^22^ investigated CAIX expression in 59 ECs and did not find a correlation. Again, possibly this study was underpowered due to a limited sample size and low number of distant recurrences. Also, differences in study design (case–control study) hamper valid comparison with our results. In other cancer types, including breast carcinoma, hepatocellular carcinoma, cervical carcinoma and renal cell carcinoma, CAIX expression is associated with poor prognosis.^33–37^ More specifically, increased distant failure was seen in several solid tumour types with positive CAIX expression.^37,38^

The metastatic process is a complex step-wise process, including acquisition of an aggressive phenotype, invasion in surrounding tissues and blood vessels, survival in the circulation with subsequent extravasation and colonisation in new organs.^39^ Hypoxia and subsequent neoangiogenesis will intervene with several steps of this process, including promoting tumour cell survival by acquisition of a malignant phenotype and increased invasion in blood vessels.^9^

A recent meta-analysis has shown that high MVD was associated with several poor prognostic variables, including deep MI, positive LVSI and poor outcome in EC, although heterogeneity due to differences in used antibodies and cut-off values hampers interpretation of these results.^23^ Biologically, intratumoural neoangiogenesis in response to hypoxia will promote the formation of vasculature with high degrees of permeability and potential for rapid growth.^40^ Our hypothesis that CAIX expression with high degrees of vascular density would be associated with unfavourable prognostic features and poor outcome was based both on the facilitation of haematogenous spread in areas with high angiogenesis and on the aggressive biological behaviour of tumour cells after hypoxia.^41,42^ HIF-1α is stabilised and accumulates under hypoxia, and activates transcription of numerous genes involved in angiogenesis, proliferation and pH regulation (VEGF, CAIX, glucose transporter-1).^9^ Our hypothesis was supported by the fact that ECs with positive CAIX expression and high vascular density had a decreased DSS compared to ECs with only one or none of both features. This observation supports the complex interplay underlying the metastatic processes. The observation that CAIX-positive ECs with high vascular density did not have more lymph node metastasis or local recurrences, but instead have more distant recurrences, could support the role of angiogenesis in the haematogenous rather than the lymphogenic metastatic process.

The obvious strengths of this study are the inclusion of a large and representative cohort of EC patients within the ENITEC network and the objective and reproducible measurement of CAIX and MVD using digital imaging analyses. However, there are some limitations that need to be addressed. Due to the retrospective nature of the study, there were missing values, specifically for LVSI and lymph node metastasis. Substantial LVSI is a stronger predictor for prognosis of EC compared to moderate LVSI. Also, LVSI is not routinely reported in the pathologic report at all centres. Therefore, we assumed that if substantial LVSI was present, it was reported, and if LVSI was not reported, no substantial LVSI was present.^31^ Missing cases were therefore coded as negative for LVSI. Separate analyses of patients with available LVSI status did not alter the results of the primary outcome (data not shown). Another general limitation in interpretation of CAIX and MVD is the lack of standardised criteria in the current literature, which hampers comparison of previous studies and this study.^29,30^ However, the applied digital techniques in this study enable objective and reproducible analyses without the need for extensive pathological expertise. With the integration of digital pathology into clinical practice, comparison of future studies with our results might be easier.^43,44^ Although widely used to quantify MVD, CD34 is known to also identify lymph vessels and stem cell populations, which theoretically could have led to an overestimation of our results. On the other hand, other antibodies, for example, CD31, also carry the risk of aspecific staining. Compared to CD31, CD34 staining is known to have stronger reactivity with endothelial cells, resulting in a lower risk of staining failure.^45^ Finally, generalisability to non-endometrioid subtypes can be questioned, as they comprise only 3.4% of the entire cohort. More research focused on this specific subgroup could help to strengthen these results.

This study identifies a group of patients with a poor DSS and DDFS based on CAIX and MVD. Given the increased risk of distant metastases, differential adjuvant treatment for these ECs could be explored either in the form of chemotherapy or, in the future, targeted therapies directed against angiogenesis. Because of the focal character of CAIX expression in the tumour tissue, performing the analysis on preoperative biopsies might be challenging, but visualisation of hypoxia and angiogenesis on FDG-PET/CT (fluorodeoxyglucose-positron emission tomography/computed tomography) scan and magnetic resonance imaging could be an alternative, as Berg et al.^5^ showed recently.

In summary, we have found that CAIX expression and high vascular density are prognostic markers for decreased survival in EC. Combining these two markers revealed that ECs with positive CAIX expression and high vascular density have an impaired outcome compared to ECs that have only one or none of both features. These patients experienced more distant recurrences, and therefore differential adjuvant treatment for these tumours should be explored.

## Supplementary information

Supplementary tables

## Data Availability

The datasets used during the current study can be made available from the corresponding author on reasonable request.
