# Supplementary material for: Poor outcome in hypoxic endometrial carcinoma is related to vascular density
Source: Br J Cancer. 2019 Apr 23;120(11):1037–44. doi: 10.1038/s41416-019-0461-2 (PMC6738053; doi:10.1038/s41416-019-0461-2)
Supplement: Supplementary file 1 — Supplementary tables [file 41416_2019_461_MOESM1_ESM.docx]

**Supplementary Table 1.** Baseline characteristics of all included patients, associated with CAIX expression.

| Variable | All (n=385) | CAIX negative (n=279) | CAIX positive (n=106) | P^a^ |
| --- | --- | --- | --- | --- |
| Age (years)^b^ | 64.0 (34.0 – 89.0) | 63.0 (34.0 – 89.0) | 66.0 (43.0 – 88.0) | 0.476 |
| Follow-up (months)^b^ | 58.0 (0.0 – 156.0) | 57.0 (0.0 – 143.0) | 62.0 (0.0 – 156.0) | 0.209 |
| Grade  Low  High | 318 (82.6)  67 (17.4) | 238 (85.3)  41 (14.7) | 80 (75.5)  26 (24.5) | 0.023 |
| Histology  EEC  NEEC | 372 (96.6)  13 (3.4) | 274 (98.2)  5 (1.8) | 98 (92.5)  8 (7.5) | 0.005 |
| Microvessel density | 262.0 (0 – 1841) | 246.0 (0 - 1221) | 297 (9 – 1841) | 0.035 |
| FIGO stage  I-II  III-IV | 363 (93.8)  22 (5.7) | 265 (95.0)  14 (5.0) | 98 (92.5)  8 (7.5) | 0.340 |
| Myometrial invasion  <50%  ≥50% | 258 (67.2)  126 (32.8) | 191 (68.7)  87 (31.3) | 67 (63.2)  39 (36.9) | 0.305 |
| LVSI^c^  No  Yes | 243 (62.8)  30 (7.8) | 175 (90.7)  18 (9.3) | 68 (85.0)  12 (15.0) | 0.172 |
| Lymph nodes^d^  No metastasis  Metastasis | 263 (68.0)  12 (3.1) | 198 (96.6%)  7 (3.4) | 65 (92.9)  5 (7.1) | 0.187 |
| Adjuvant treatment  No  Radiotherapy  Chemotherapy | 154 (40.0)  200 (51.9)  31 (8.1) | 125 (44.8)  136 (48.7)  18 (6.5) | 29 (27.4)  64 (60.4)  13 (12.3) | 0.041  0.067 |
| Recurrence  No  Yes  Local  Regional  Distant | 338 (87.8)  47 (12.2)  14 (3.6)  16 (4.2)  31 (8.1) | 250 (89.6)  29 (10.4)  11 (3.9)  11 (3.9)  17 (6.1) | 88 (83.0)  18 (17.0)  3 (2.8) 5 (4.7)  14 (13.2) | 0.078  0.766  0.734  0.022 |
| Death  No  Yes  EC-related | 335 (87.0)  50 (13.0)  21 (5.5) | 251 (90.0)  28 (10.0)  10 (3.6) | 84 (79.2)  22 (20.8)  11 (10.4) | 0.005  0.009 |

*^a^P-value of the Mann-Whitney U test for continuous, and χ^2^ test and Fishers’ exact for categorical variables  ^b^Median values (range),^c^based on 273 patients, ^d^based on 275 patients, EEC, endometrioid endometrial carcinoma; NEEC, non-endometrioid endometrial carcinoma; FIGO, International Federation of Gynecology and Obstetrics; LVSI, Lymphovascular Space Invasion; EC, Endometrial carcinoma*

| DSS | HR (95% CI) | P | HR (95% CI) | P |
| --- | --- | --- | --- | --- |
| Age (years)  ≤65 - >65 | 3.74 (1.48 – 9.49) | 0.005 | 2.59 (1.00 – 6.72) | 0.051 |
| CAIX  Negative-positive | 3.20 (1.40 – 7.31) | 0.006 | 2.45 (1.05 – 5.73) | 0.039 |
| Grade  Low-High | 6.39 (2.81 – 14.53) | <0.001 | 2.87 (1.08 – 7.60) | 0.034 |
| Myometrial invasion  <50% - ≥50% | 3.22 (1.39 – 7.43) | 0.006 | 1.38 (0.52 – 3.67) | 0.525 |
| FIGO-stage  I/II-III/IV | 7.35 (2.86 – 18.88) | <0.001 | 2.89 (0.52 – 3.67) | 0.094 |
| LVSI  No – yes | 5.58 (2.30 – 13.56) | <0.001 | 1.58 (0.49 – 5.11) | 0.443 |

**Supplementary table 2.** Univariable and multivariable analysis of clinicopathological parameters including CAIX-expression as prognosticators of DSS

*DSS, disease specific survival; CI, confidence interval.*

**Supplementary Table 3.** Baseline characteristics of all included patients, associated with MVD.

| Variable | MVD < median (n=195) | MVD > median (n=190) | P^a^ |
| --- | --- | --- | --- |
| Age (years)^b^ | 64.0 (41.0 – 88.0) | 64.0 (41.0 -89.0) | 0.742 |
| Follow-up (months)^b^ | 59.0 (1.0 – 156.0) | 57.0 (0.0 – 156.0) | 0.245 |
| Grade  Low  High | 160 (82.1)  35 (17.9) | 158 (83.2)  32 (16.8) | 0.790 |
| Histology  EEC  NEEC | 189 (96.9)  6 (3.1) | 183 (96.3)  7 (3.7) | 0.742 |
| FIGO stage  I-II  III-IV | 182 (93.3)  13 (6.7) | 181 (95.3)  9 (4.7) | 0.415 |
| Myometrial invasion  <50%  ≥50% | 140 (72.2)  54 (27.8) | 118 (62.1)  72 (37.9) | 0.036 |
| LVSI^c^  No  Yes | 182 (93.3)  13 (6.7) | 173 (91.1)  17 (8.9) | 0.404 |
| Lymph nodes^d^  No metastasis  Metastasis | 130 (93.5)  9 (6.5) | 133 (97.8)  3 (2.2) | 0.083 |
| Adjuvant treatment  No  Radiotherapy  Chemotherapy | 82 (42.1)  98 (50.3)  15 (7.7) | 72 (37.9)  102 (53.7)  16 (8.4) | 0.501  0.793 |
| Recurrence  No  Yes  Local  Regional  Distant | 170 (87.2)  25 (12.8)  8 (4.1)  10 (5.1)  14 (7.2) | 168 (88.4)  22 (11.6)  6 (3.2)  6 (3.2)  17 (8.9) | 0.710  0.621  0.333  0.524 |
| Death  No  Yes  EC-related | 173 (88.7)  22 (11.3)  6 (3.1) | 160 (84.2)  30 (15.8)  17 (8.9) | 0.196  0.015 |

*^a^P-value of the Mann-Whitney U test for continuous, and χ^2^ test and Fishers’ exact for categorical variables  ^b^Median values (range),^c^based on 273 patients, ^d^based on 275 patients, EEC, endometrioid endometrial carcinoma; NEEC, non-endometrioid endometrial carcinoma; FIGO, International Federation of Gynecology and Obstetrics; LVSI, Lymphovascular Space Invasion; EC, Endometrial carcinoma*

**Supplementary Table 4.** Univariable and multivariable analysis of clinicopathological parameters including MVD as prognosticators of DSS.

| DSS | HR (95% CI) | P | HR (95% CI) | P |
| --- | --- | --- | --- | --- |
| Age (years)  ≤65 - >65 | 3.74 (1.48-9.49) | 0.005 | 3.12 (1.21-8.02) | 0.019 |
| MVD  Negative-positive | 3.20 (1.40-7.31) | 0.006 | 2.92 (1.13-7.54) | 0.027 |
| Myometrial invasion  <50% - ≥50% | 3.22 (1.39-7.43) | 0.006 | 1.11 (0.42-2.97) | 0.835 |
| FIGO-stage  I/II-III/IV | 7.35 (2.86-18.88) | <0.001 | 3.16 (0.83-12.07) | 0.092 |
| Grade  1/2 – 3 | 6.39 (2.81-14.53) | <0.001 | 3.30 (1.23-8.99) | 0.018 |
| LVSI  No-yes | 5.58 (2.30-13.58) | <0.001 | 1.53 (0.43-5.45) | 0.514 |
